# Elevated serotonin coordinates mammary metabolism in dairy cows

**DOI:** 10.14814/phy2.14798

**Published:** 2021-04-09

**Authors:** Meghan K. Connelly, Samantha R. Weaver, Jordan M. Kuehnl, Hannah P. Fricke, Marisa Klister, Laura Hernandez

**Affiliations:** ^1^ Department of Animal and Dairy Sciences University of Wisconsin Madison WI USA

**Keywords:** 5‐HTP, calcium, dairy cow, mammary gland, serotonin

## Abstract

Serotonin plays a diverse role in maternal and mammary metabolism. Recent research in the dairy cow has shown a relationship between serotonin and calcium, with increased serotonin concentrations improving calcium homeostasis in the peri‐partum dairy cow. Therefore, the objective was to elucidate how administration of 5‐hydroxy‐l‐tryptophan (5‐HTP), the immediate precursor to serotonin, altered serotonin and calcium metabolism in lactating dairy cows. Twelve mid‐late lactation multiparous cows were blocked by parity, production and days in milk and allocated to a daily intravenous infusion of (i) 1.5 mg/kg of 5‐HTP (*n* = 6) or (ii) saline (*n* = 6) for 3 consecutive days. Milk samples were collected daily. Blood samples were collected before and after each infusion with mammary biopsies and blood samples collected at 48, 56, and 72 h relative to termination of first infusion. Infusion of 5‐HTP increased (*p* = 0.001) circulating serotonin concentrations and decreased blood calcium via a transient hypocalcemia immediately after each infusion (*p* = 0.02). Treatment with 5‐HTP increased milk calcium concentrations (*p* = 0.02) and calcium release‐activated channel protein 1 (ORAI1) mRNA at 56 h and protein at 48 h relative to termination of first infusion (*p* = 0.008 and *p* = 0.09, respectively). Fifty‐six hours from termination of the first infusion mRNA of parathyroid hormone‐related protein and mammary serotonin content were increased relative to control (*p* = 0.03 and *p* = 0.05, respectively). These findings demonstrate the ability of 5‐HTP infusion to increase circulating serotonin concentrations and alter endocrine and mammary autocrine/paracrine calcium and serotonin metabolism in the lactating dairy cow.

## Graphical abstract

Our data demonstrates that serotonin regulates mammary calcium metabolism. The control of calcium by serotonin stimulates a negative feedback loop. This data demonstrates that the mammary gland acts via an endocrine organ to control maternal metabolism.

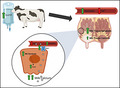

## 1 INTRODUCTION

The onset of lactation requires a variety of physiological adaptions as a mammal transitions from a non‐lactating to lactating state. In order to initiate lactation, a coordination of changes in tissue metabolism occurs in response to the physiological demands of lactation. Due to these immense nutrient demands by the mammary gland, many maternal adaptations occur to support lactation (Bauman & Currie, 1980). To meet these demands, in addition to traditionally thought of systemic signals, there is growing evidence that the mammary gland in the periparturient period functions as an endocrine gland by synthesizing and releasing hormones to initiate changes in maternal physiology needed to support lactation. Recent research has focused on exploring some of these mammary‐specific processes, such as PTHrP and serotonin, and how they may contribute to homeorhesis in the dairy cow during lactation (Horseman & Collier, 2014; Horst et al., 2005; Wysolmerski, 2012).

Serotonin plays pivotal roles in both metabolism and physiology, functioning as a neurotransmitter, vasoconstrictor, gastrointestinal health regulator, and an important mammary‐derived hormone to aid in coordination of lactation (Berger et al., 2009; Horseman & Collier, 2014). Serotonin is synthesized in a two‐step pathway, originating from the amino acid l‐tryptophan and converted to 5‐hydroxy‐l‐tryptophan (5‐HTP) by the enzyme tryptophan hydroxylase 1 (TPH1) in the periphery (Walther & Bader, 2003). After conversion to 5‐HTP, decarboxylation occurs by aromatic amino acid decarboxylase to produce serotonin. Serotonin is metabolized by one of two major forms of monoamine oxidase (MAO), with the primary inactivation occurring by MAO‐A (Mohammad‐Zadeh et al., 2008). Serotonin is classically considered a neurotransmitter, but neuronal serotonin is considered a separate entity from peripheral serotonin, as each are incapable of passing the blood‐brain barrier (Walther & Bader, 2003).

Serotonin is also a potent regulator of mammary homeostasis, with ~50% of circulating serotonin being synthesized and secreted from mammary epithelial cells (MEC) during lactation (Weaver et al., 2017). In a non‐lactating state, the majority of serotonin is synthesized and secreted by enterochromaffin cells within the intestine (Gershon & Tack, 2007). Recently, research has focused on serotonin's influence on calcium homeostasis during lactation in the rodent and the dairy cow (Laporta, Keil, Vezina et al., 2014; Laporta, Keil, Weaver, et al., 2014; Laporta et al., 2015; Slater et al., 2018). Mammary‐derived serotonin stimulates synthesis and secretion of parathyroid hormone related protein (PTHrP) from the mammary gland into circulation (Hernandez et al., 2012; Laporta, Keil, Vezina et al., 2014; Laporta, Keil, Weaver, et al., 2014; Laporta et al., 2013). Upon entering circulation, PTHrP acts on bone to enable calcium mobilization to meet the demands for milk calcium while maintaining maternal normocalcemia (Wysolmerski, 2010, 2012). However, most research on serotonin's regulation of mammary calcium metabolism has been in rodents, with minimal evidence available for understanding this biological mechanism in the dairy cow.

Previous research has demonstrated that administration of 5‐HTP, the immediate precursor to serotonin, alters circulating serotonin and calcium concentrations, therefore posing a novel approach for hypocalcemic mitigation in the dairy industry. Dairy cows are uniquely challenged during lactation to maintain calcium homeostasis due to copious milk production, with a blood calcium pool turnover of 20–30 times per day during peak production (Horst et al., 2005). When cows were infused with 5‐HTP pre‐partum, a corresponding increase in circulating serotonin also coincided with an improvement in post‐partum calcium metabolism (Slater et al., 2018; Weaver et al., 2016). However, a thorough understanding of how serotonin signals and acts within the dairy cow and at the level of the mammary gland has yet to be clearly illustrated.

The objective of this study was to determine how 5‐HTP infusion over a 3d period in lactating Holstein cows altered calcium and serotonin metabolism locally and systemically. We hypothesized that 5‐HTP infusion would alter blood serotonin and elicit a transient hypocalcemia. Further, we hypothesized that these blood calcium and serotonin changes would modulate milk calcium concentrations and genes involved in calcium and serotonin metabolism in the mammary gland.

## 2 MATERIALS AND METHODS

### 2.1 Animals and experimental design

All experimental procedures and guidelines (A005903) in this study were approved by the College of Agriculture and Life Sciences Animal Care and Use Committee at the University of Wisconsin‐Madison and strictly followed. Cows were housed in a tie‐stall barn at the Dairy Cattle Center at the University of Wisconsin‐Madison, milked twice daily and fed the standard herd lactating cow diet (Vita Plus, Lake Mills, WI, USA). Sample size was calculated to provide 80% power with an alpha of 0.05 to be able to detect a 0.3 mM decrease in blood calcium concentrations in response to 5‐HTP treatment. In case of animal removal, an additional animal was added which resulted in an *n* = 6 per treatment.

**FIGURE 1:**
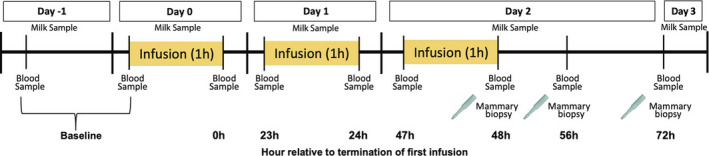
Schematic representation of experimental design and sampling timeline. Twelve mid‐late lactation multiparous Holstein dairy cows were blocked by parity, milk production and days in milk in a randomized complete block design and assigned to intravenous infusion of (i) 1 L of 1.5 mg/kg 5‐HTP dissolved in saline (*n* = 6/treatment) or (ii) 1 L of saline solution (*n* = 6/treatment) for three consecutive days. Milk samples were collected daily at the morning milking. Day 0 milk samples were taken prior to treatment administration.

Twelve multiparous Holstein dairy cows (212.17 ± 20.04 days in milk; 2.5 ± 0.26 average lactation parity, 41.21 ± 2.31 kgs/d average milk yield) were blocked by parity, milk production and days in milk in a randomized complete block design and assigned to intravenous infusion of (i) 1 L of 1.5 mg/kg 5‐HTP dissolved in saline (*n* = 6/treatment) or (ii) 1 L of saline solution (*n* = 6/treatment) for three consecutive days. Infusions were administered via jugular catheter at a constant rate over 1‐h periods. Infusions of 5‐HTP (H9772, Sigma‐Aldrich) were calculated on a mg/kg of BW basis for each cow with 5‐HTP mixed in saline until dissolved, and then sterile filtered and stored at 4°C. Baseline blood samples were collected one day prior to administration of treatment. Blood samples were collected prior to and immediately after administration of infusion on d0, d1 and d2 of the period. Additional blood samples and mammary biopsies were taken immediately after (48 h relative to termination of first infusion), 8 h (56 h relative to termination of first infusion) and 24 h (72 h relative to termination of first infusion) after termination of third infusion. Milk samples were collected daily at the morning milking (Figure 1) with infusion administration occurring after milk sample collection on d0. Therefore, d0 was used as part of the covariate in milk calcium and serotonin analysis.

### 2.2 Sample collection

Prior to start of treatment, a single jugular catheter was inserted under IV sedation with 0.02 mg/kg xylazine (Vedco) and maintained as previously described (Slater et al., 2018). Mammary biopsies were performed at 48, 56 and 72 h relative to termination of first infusion with sedation, surgical preparation, surgical technique and closure as previously described (Slater et al., 2018). Mammary tissues collected were rinsed in 1 × PBS and divided for respective lab analysis. Tissues used for RNA and protein were immediately snap‐frozen in liquid nitrogen and stored at −80°C. Tissues collected for histology were fixed in 4% paraformaldehyde overnight at 4°C and then transferred and stored in 70% ethanol until paraffin embedding. Sufficient mammary tissue was not always obtained at each timepoint for all three analyses from each cow due to biopsy limitations. Biopsy sites were alternated between the two rear quarters of the cow. Cows were monitored at catheter and biopsy sites daily with rectal temperatures recorded.

Baseline blood samples were collected via coccygeal vein. Prior to whole blood collection from the jugular, 8 ml of blood were drawn then discarded with whole blood then collected from the catheter immediately before and after infusion on d0, d1 and d2 with additional sampling occurring at eight (56 h) and 24 h (72 h) after termination of the third infusion. Whole blood was collected into 10 ml BD Vacutainer K2 EDTA Plus (368589, BD), 10 ml BD Vacutainer Serum (367820, BD) and 10 ml Lithium Heparin 158 USP Units (367880, BD) blood collection tubes. Immediately following inversions, 3–4 ml of whole blood was transferred from the 10 ml BD Vacutainer K_2_ EDTA Plus tube into an Eppendorf preloaded with ascorbic acid (Connelly et al., 2020; Vatassery et al., 1981) and frozen at −20°C. Serum samples were allowed to clot at room temperature, then along with plasma samples were isolated from vacutainer tubes by centrifugation at 3000*g* for 20 min at 4°C. Respective blood fractions were aliquoted and stored at −80°C.

Milk samples were collected daily at the morning milking starting one day prior to the first infusion through the morning after the last infusion. Milk samples were collected in 5 ml Eppendorf tubes and stored at −80°C.

### 2.3 Whole blood, serum, plasma and milk laboratory analyses

Serotonin concentrations were analyzed in whole blood and milk via Serotonin EIA (IM1749, Immunotech, Beckman Coulter). Whole blood samples were diluted 1:100 and milk diluted 1:5 to fit within the standard curve. The intra‐ and inter‐assay coefficients of variation (CV) for blood serotonin were 2.47% and 10.49%, respectively, with milk serotonin 8.62% and 4.63%, respectively. Total serum calcium concentrations were determined using a colorimetric assay (700550, Cayman Chemical). Serum calcium samples were diluted 1:2 to fit within the standard curve. The intra‐ and inter‐assay CVs were 2.79% and 3.99%, respectively. Milk calcium concentrations were determined by colorimetric assay (DICA‐500, QuantiChrom Calcium Assay). Milk calcium samples were diluted 1:25 to fit within the standard curve. The intra‐ and inter‐assay CVs were 4.80% and 6.65%, respectively. Milk protein concentrations were determined by Bicinchoninic Acid Assay (23227, Pierce Chemicals) in order to correct for casein‐bound calcium (Mamillapalli et al., 2013) and calculated by dividing milk calcium mM by milk protein. Samples were diluted 1:100 to fit within the standard curve. The intra‐ and inter‐assay CVs were 2.38% and 1.16%, respectively. Parathyroid hormone (PTH) concentrations were analyzed in plasma using Bovine IPTH (Intact PTH) ELISA Kit (DEIA1826B, Creative Diagnostics). Plasma PTH samples were diluted 1:2 to fit within the standard curve. The intra‐ and inter‐assay CVs were 3.51% and 11.49%, respectively.

### 2.4 Mammary gland gene expression

Total RNA was extracted from mammary tissue using TRI‐Reagent (NC9277980, Molecular Research, Thermo Fisher Scientific) according to manufacturer's instructions. Total RNA concentration was determined by quantification of absorbance ratios by Nanodrop spectrophotometer (ND‐1000, Nanodrop Technologies). One µg of RNA was reverse transcribed to cDNA using the Applied Biosystems high‐capacity cDNA Reverse Transcription Kit (4368814, Applied Biosystems) and diluted (1:5) in molecular grade water. qRT‐PCR was performed using the CFX96 Touch Real‐Time PCR Detection System (Bio‐Rad Laboratories). Mammary expression of genes involved in serotonin and calcium metabolism (calcium release activated channel protein 1, *ORAI1*; plasma membrane calcium ATPase 2, *PMCA2*; ATPase secretory pathway calcium transporting 1, *SPCA1*; ATPase sarcoplasmic/endoplasmic reticulum calcium transporting 2, *SERCA2*; solute carrier family 8 membrane 1, *NCX1*; parathyroid hormone related protein, *PTHLH*; calcium sensing receptor, *CASR*; serotonin receptor 2b, *5HTR2b*; serotonin receptor 7, *5HTR7*; serotonin reuptake transporter, *SERT*; *MAO*‐*A*; *TPH1*) were evaluated using primer sequences supplied in Table 1. Reaction mixtures, cycling conditions, and primer design were performed as previously described (Slater et al., 2018). The geometric mean of keratin 8 (*K8*), ribosomal protein 9 (*RPS9*), and cyclophilin A (*CYCLOA*) were used as the housekeeping parameter. Data were analyzed using the 2^−∆∆Ct^ method with saline infused (control) cows serving as the internal control at each timepoint (Livak & Schmittgen, 2001).

**TABLE 1:** Primer sequences used for quantitative real‐time PCR performed in mid‐late lactation multiparous Holstein dairy cows. All primers were designed and sequences obtained as in Slater et al., 2018. Reactions were run at an annealing temperature of 60°C. The geometric mean of *K8*, *RSP9* and *CYCLOA* were used as the housekeeping parameter

Genes	Forward Primer 5'−3’	Reverse Primer 3'−5’
*ORAI1*	GGCGCAAACTCTACTTGAGC	GGTAGTCGTGGTCAGCATCC
*PMCA2*	CATCAAGTGTGGCATCATCC	TGGCCAGATCTTATCGATCC
*SPCA1*	TGCTCTTGCAATGAAGATGG	CGGTGCACACACTTAACAGC
*SERCA2*	TTCCGTTACCTGGCTATTGG	ATTCAAAGACCGCACAATCC
*NCX1*	TGTGGCCATAACTTCACTGC	ACGCAGATGCTTGATCTTCC
*PTHLH*	GGAGGCTAGTTCAGCAATGG	CCGAGGTAGCTCTGATTTCG
*CASR*	ACACGTGGTTCCAAGAGAGC	CAGCAGTATGCCATTCAACG
*TPH1*	AGAGAATTTACCAAGACAATCAAG	CTTAGCAAGGGCATCACTGAC
*5HTR2b*	CTGGCTTCCTTCTTCACACC	AACCATGTTAGGCGTTGAGG
*5HTR7*	AATCATTTGCCGAGACTTCG	CGGATCCACAGAAAACAAGG
*MAO‐A*	CATCGATAACTGCCCTGTGG	ATTGCACGGCTGTTCTATGG
*SERT*	GAAGCTGTTGGAGGAGTTCG	CCAGCAGATCTTCCAGAACC
*K8*	GATGAACCGGAACATCAACC	GCCTGACATCCTTAACAGC
*RPS9*	GGAGACCCTTCGAGAAGTCC	CTTTCTCATCCAGCGTCAGC
*CYCLOA*	CACCGTGTTCTTCGACATCG	ACAGCTCAAAAGAGACGCGG

Abbreviations: 5HTR2b, serotonin receptor 2b; 5HTR7, serotonin receptor 7; CASR, calcium sensing receptor; CYCLOA, cyclophilin A; K8, keratin 8; MAO‐A, monoamine oxidase A; NCX1, solute carrier family membrane 1; ORAI1, calcium release activated channel protein 1; PMCA2, plasma membrane calcium ATP‐ase 2; PTHLH, parathyroid hormone related protein; RPS9, ribosomal protein 9; SERCA2, ATPase sarcoplasmic/endoplasmic reticulum calcium transporting 2; SERT, serotonin reuptake transporter; SPCA1, ATPase secretory pathway calcium transporting 1; TPH1, tryptophan hydroxylase 1.

### 2.5 Protein isolation and analysis

Protein was isolated from mammary tissue using radio‐immunoprecipitation assay (RIPA) buffer for serotonin content and Triton lysis buffer for calcium content in addition to 10 µl/ml of Halt Protease and Phosphatase Inhibitors Cocktail (78441, Thermo Scientific). Protein concentrations were determined by Bicinchoninic Acid Assay (20831001‐1, Bioworld). Mammary gland serotonin content was determined using the Serotonin EIA Kit (IM1749, Immunotech, Beckman Coulter) using 50 µg of protein per sample as previously analyzed and reported (Laporta et al., 2015). Mammary gland calcium content was determined using (700550, Cayman Chemical) 83.3 µg of protein per sample per well.

### 2.6 Western blotting

Protein lysates were diluted with 5× sample buffer containing SDS and ß‐mercaptoethanol and heated at 95°C for 10 min. Mammary gland protein extracts (*n* = 4 per treatment, 20 µg) were separated by electrophoresis on a gradient (8%–20%) SDS‐polyacrylamide gel and transferred for 1 h at 100 V onto a polyvinylidene difluoride membrane (IPVH00010, Millipore Sigma). The membrane was blocked with Sea Block Blocking Solution (Thermo Fisher Scientific) and probed at 4°C overnight with 1:1000 ORAI1 (MA5‐15776, Thermo Fisher Scientific) and 1:1000 ß‐actin (4967A; Cell Signaling). The following day, the membrane was washed with TBST and probed 1:5000 with fluorescent secondary antibodies (925–32213, IRDye 800 CW; 925–68070, IRDye 680, Li‐Cor Biosciences). Protein bands were identified using Li‐Cor Odyssey Fc (Li‐Cor) with a 2‐min exposure for 700 channel and 10‐min exposure for 800 channels. Analysis of images and protein band quantification were conducted using the Image Studio Lite software (Li‐Cor Biosciences, version 5.2).

### 2.7 Mammary gland histology

Mammary tissue sections (5 µm) were deparaffinized and stained with hematoxylin and eosin. Two non‐overlapping images were taken, and alveolar quantity and size were quantified using the ImageJ software (Version 1.8.0, National Institute of Health) according to previous literature (Skibiel et al., 2018). Alveolar quantity and size values were averaged between the two images and then statistically analyzed (48 h, *n* = 5; 56 h, *n* = 5; 72 h, *n* = 3).

### 2.8 Statistical analysis

Data were analyzed using the MIXED procedure of SAS (version 9.4, SAS Institute Inc.). Fixed terms in the model for blood variables included treatment, block, covariate, time, and treatment*time. Covariate was a means of baseline blood samples taken prior to treatment administration. Fixed terms in the model for protein, mRNA, histological findings, and mammary gland content included treatment, block, time, and treatment*time. Hour was considered the repeated measure, and in order to account for autocorrelated errors, the spatial power structure was used within the SAS mixed procedure. Fixed terms within the model for milk yield, milk calcium, and milk serotonin were treatment, block, covariate, day, and treatment*day. Covariate values were means of baseline samples taken prior to treatment administration. Day was considered the repeated measure, and autocorrelated errors were accounted for utilizing the ar(1) structure. The random statement in all models included cow (block). Data were analyzed for normality, and when that assumption failed, data were transformed and analyzed. Transformations were based on diagnostic plots and overall model fit with either rank or log transformation then performed on response variables. Studentized residuals of models were evaluated and if values greater than three arose observations were identified as outliers and removed (Chatterjee & Hadi, 2015; Cook, 1977). Visual presentation of transformed data is presented as mean ±SEM. Normally distributed data are presented as LSMEANS ± SEM. Statistical significance was declared at *p ≤ *0.05.

## 3 RESULTS

### 3.1 Milk yield, alveolar quantity, and area were not altered by 5‐HTP infusion

**FIGURE 2:**
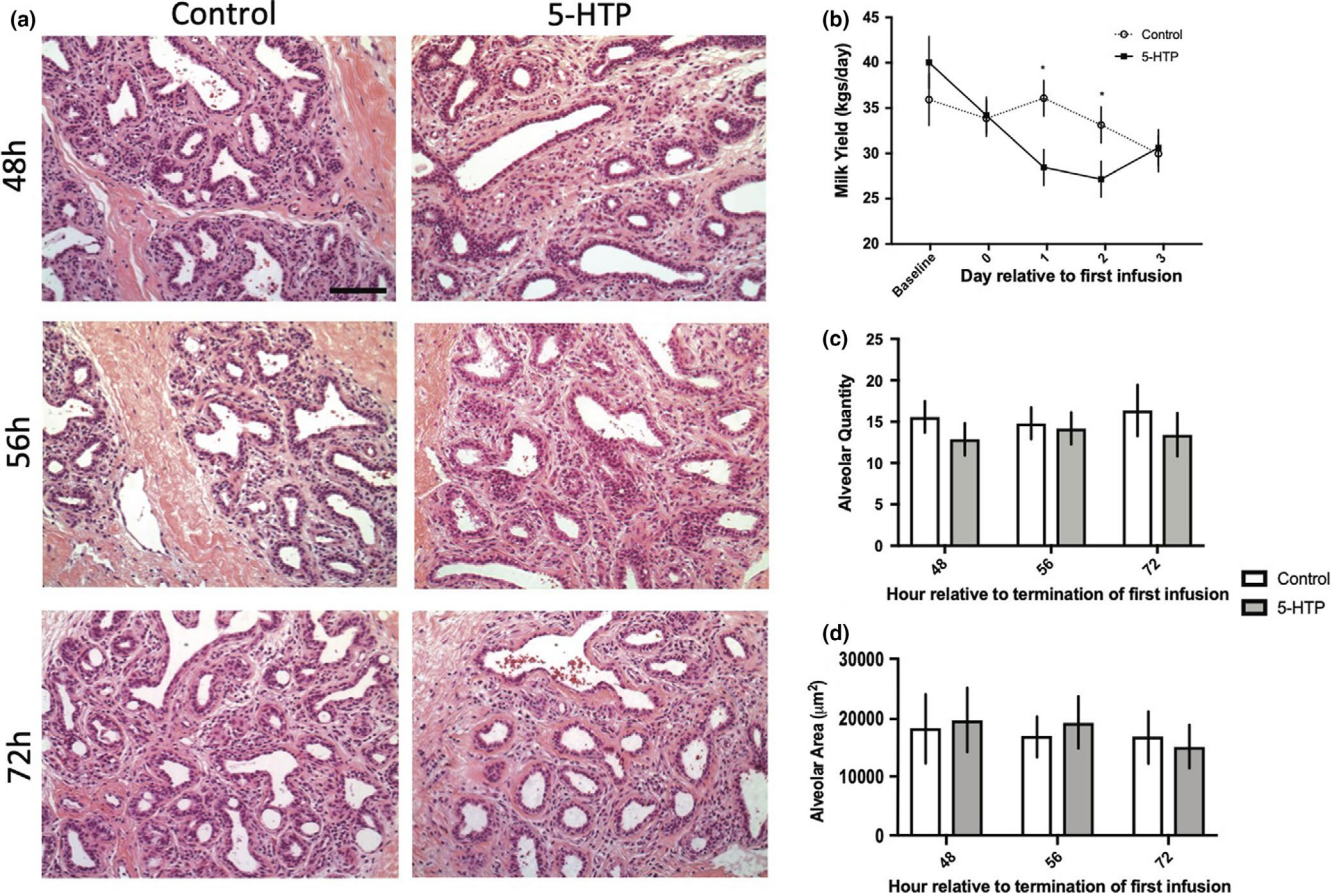
Mammary gland histological findings and milk yield (kgs/d) in multiparous Holstein dairy cows receiving 1 L of 1.5 mg/kg 5‐HTP dissolved in saline or 1 L of saline solution (Control = Saline) for three consecutive days. Mammary gland tissues were sectioned (5 µm) and stained with hematoxylin and eosin (a) and analyzed with Image J software to determine alveolar quantity and area (c–d) (48 h *n* = 5; 56 h *n* = 5; 72 h *n* = 3). Images were taken at 20x magnification (scale bar: 100 µm). Milk yield of 5‐HTP and control cows across the experimental period (b) (*n* = 6). Statistics were performed using PROC MIXED procedure in SAS with repeated measures. Data presented as LSMEANS ± SEM. Milk yield: day (*p* = 0.01) and treatment*day (*p* = 0.008). Asterisks denote statistical significance (*p < 0.05)

Under conditions of milk stasis, supraphysiological action of serotonin may act on mammary epithelial cells to reduce milk secretion (Horseman and Collier, 2014; Stull et al., 2007). Therefore, we evaluated potential implications on performance and mammary tissue. There was no difference in overall milk yield between 5‐HTP and control cows (*p* > 0.05). A significant effect of day occurred, with milk production decreasing across the experimental period in all animals irrespective of treatment (*p* = 0.01). A treatment by day effect was observed with control cows having higher milk yield on d1 and d2 of treatment (*p* = 0.008), but no differences in milk yield occurred on d3 of the experimental period between treatments (Figure 2b). No differences (*p* > 0.05) were detected between treatments in alveolar quantity and area (Figure 2c–d). While milk yield differences occurred within day, no treatment differences occurred across the experimental period or in alveolar quantity and area.

### 3.2 Circulating serotonin was increased in response to 5‐HTP administration

**FIGURE 3:**
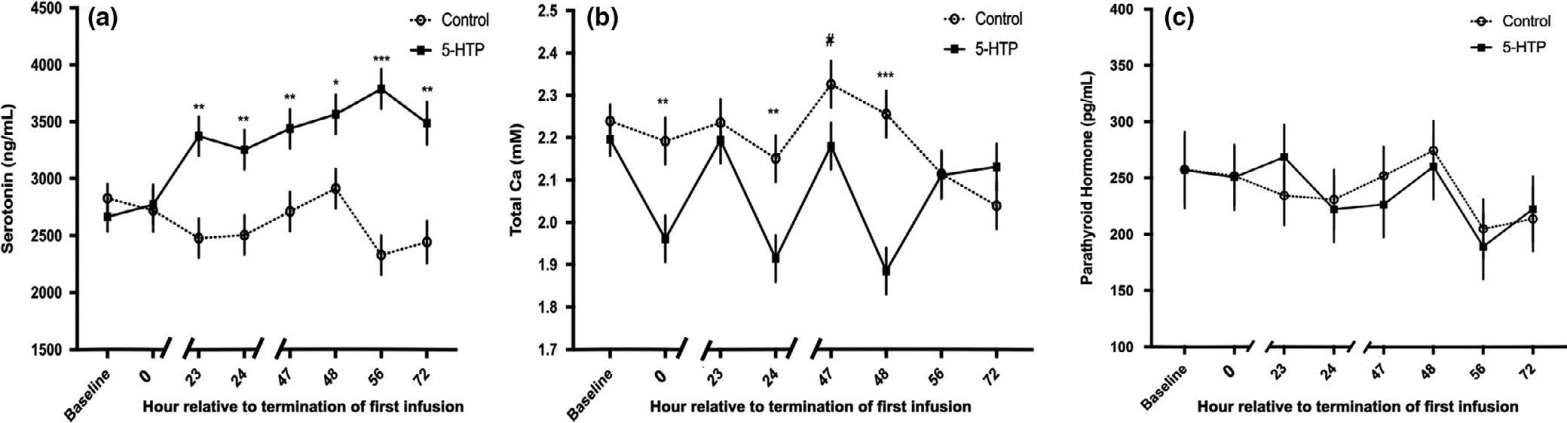
Whole blood serotonin concentrations (a) (*n* = 6), serum total calcium concentrations (b) (*n* = 6) and plasma parathyroid hormone concentrations (c) (5‐HTP *n* = 5, Control *n* = 6) in multiparous Holstein dairy cows receiving 1 L of 1.5 mg/kg 5‐HTP dissolved in saline or 1 L of saline solution (Control = Saline) for three consecutive days. Statistics were performed using PROC MIXED procedure in SAS with repeated measures. Data presented as LSMEANS ± SEM. Whole blood serotonin concentrations: treatment (*p* = 0.001), time (*p* = 0.07) and treatment*time (*p* = 0.004). Serum total calcium concentrations: treatment (*p* = 0.02), time (*p* < 0.0001) and treatment*time (*p* = 0.0002). Asterisks denote statistical significance between groups at each timepoint (***p < 0.001, **p < 0.01, *p < 0.05, and ^#^0.10 < *p* > 0.05)

Administration of 5‐HTP increased circulating serotonin concentrations in 5‐HTP cows across the experimental period (*p* = 0.001). Interestingly, no difference in blood serotonin concentrations were detected immediately after termination of infusion on d0 of treatment (*p* > 0.05; Figure 3a). However, whole blood serotonin concentrations were increased in 5‐HTP cows 23 h after termination of the first infusion, with elevation maintained for the remainder of the experimental period (*p* < 0.05). Circulating serotonin concentrations increased across time (*p* = 0.06), and an interaction between treatment and time was observed (*p* = 0.004), with blood serotonin concentrations continuing to increase across the experimental period in 5‐HTP infused cows.

### 3.3 5‐HTP infusion decreased blood calcium concentrations and did not alter plasma PTH

Blood calcium concentrations were decreased in 5‐HTP cows relative to control across the experimental period (*p* = 0.02). Immediately after each daily infusion, a transient hypocalcemia occurred resulting in decreased blood calcium concentrations in 5‐HTP cows (1.92 ± 0.05 mM), with an effect of both time (*p* < 0.0001) and treatment by time (*p* = 0.0002). Greater subsequent declines in blood calcium occurred with each 5‐HTP infusion, with the lowest blood calcium concentration occurring immediately after the final infusion on d2 of the experimental period (1.88 ± 0.05 mM; *p* < 0.0001; Figure 3b). Eight hours after termination of the final 5‐HTP infusion (56 h), 5‐HTP infused cow's blood calcium concentrations were normocalcemic and were not different relative to control (*p* > 0.05; Figure 3b). Interestingly, PTH, a fundamental calciotropic hormone, was unresponsive to infusion of 5‐HTP (Figure 3c), despite a significant blood calcium decline immediately after 5‐HTP infusion.

### 3.4 5‐HTP infusion alters milk calcium and calcium metabolism genes in the mammary gland

**FIGURE 4:**
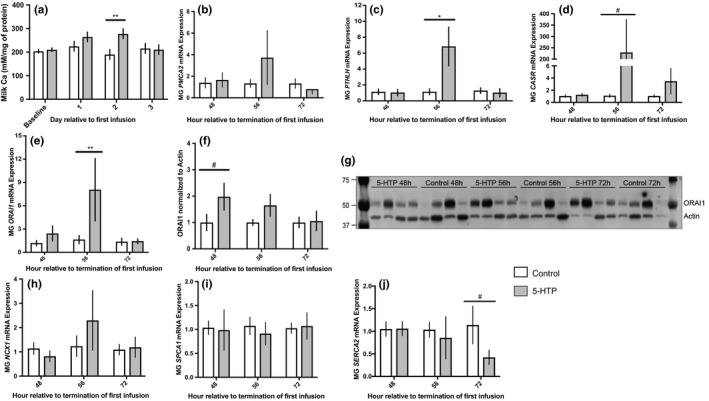
Milk calcium concentrations (a) (*n* = 6), mammary gland mRNA expression of *PMCA2* (b) (5‐HTP *n* = 6, *n* = 6, *n* = 5; Control *n* = 6, *n* = 6, *n* = 6), *PTHLH* (c) (5‐HTP *n* = 6, *n* = 5, *n* = 6; Control *n* = 4, *n* = 4, *n* = 5), *CASR* (d) (5‐HTP *n* = 6, *n* = 5, *n* = 5; Control *n* = 3, *n* = 4, *n* = 3), *ORAI1* (e) (5‐HTP *n* = 6, *n* = 6, *n* = 6; Control *n* = 5, *n* = 6, *n* = 6), quantification and western blot of mammary gland ORAI1 (f–g) (5‐HTP *n* = 4, *n* = 4, *n* = 3; Control *n* = 4, *n* = 4, *n* = 3), mammary mRNA expression of *NCX1* (h) (5‐HTP *n* = 6, *n* = 6, *n* = 6; Control *n* = 6, *n* = 5, *n* = 5), *SPCA1* (i) (5‐HTP *n* = 6, *n* = 6, *n* = 6; Control *n* = 5, *n* = 6, *n* = 5), and *SERCA2* (j) (5‐HTP *n* = 6, *n* = 6, *n* = 6; Control *n* = 5, *n* = 4, *n* = 3) in multiparous Holstein dairy cows receiving 1 L of 1.5 mg/kg 5‐HTP dissolved in saline or 1 L of saline solution (Saline = Control) for three consecutive days. Statistics were performed using PROC MIXED procedure with repeated measures in SAS. Transformed data presented as mean ± SEM. Normally distributed data presented as LSMEANS ± SEM. Milk calcium: treatment (*p* = 0.02). ORAI1 mRNA: treatment (*p* = 0.09) and treatment*time (*p* = 0.06). *PTHLH* mRNA: time (*p* = 0.04) and treatment*time (*p* = 0.03). Asterisks denote statistical significance between groups (**p < 0.01, *p < 0.05 and #0.10 < *p* > 0.05)

Cows infused with 5‐HTP had increased milk calcium concentrations compared to control cows (251.01 ± 10.69 vs. 210.22 ± 10.69 mM/mg protein, respectively; *p* = 0.02) across the duration of the experiment. Cows treated with 5‐HTP had significantly increased milk calcium relative to control cows on d2 (*p* = 0.006; Figure 4a). Infusion of 5‐HTP did not alter *PTHLH* (*p* > 0.05) mRNA across the experimental period; however, an approximate five and a half‐fold increase occurred 8 h after termination of the third infusion (56 h) in 5‐HTP treated cows (*p* = 0.03; Figure 4c). Mammary *CASR* mRNA was increased across the experiment (*p* = 0.10), mirroring *PTHLH* mRNA, with an approximate 200‐fold increase relative to control cows 8 h after termination (56 h) of the third infusion (*p* = 0.09; Figure 4d). Treatment with 5‐HTP increased *ORAI1* mRNA and protein expression throughout the experiment (*p* = 0.08 and *p* = 0.14, respectively). Mammary *ORAI1* mRNA and protein were increased post termination, with increased mRNA 8 h (56 h) after termination of final infusion and ORAI1 protein immediately (48 h) after termination of final infusion (*p* = 0.008 and *p* = 0.09, respectively; Figure 4e–g). No treatment differences were detected in mammary mRNA expression of *PMCA*2, *SERCA2*, *NCX1* or *SPCA1* (*p* > 0.05) across the experiment. Interestingly, there was decreased mRNA expression of *SERCA2* in 5‐HTP treated cows 24 h (72 h) after termination of the third infusion (*p* = 0.09; Figure 4j).

### 3.5 Mammary serotonin metabolism genes were up‐regulated after 5‐HTP administration, but milk serotonin concentrations were not significantly altered

**FIGURE 5:**
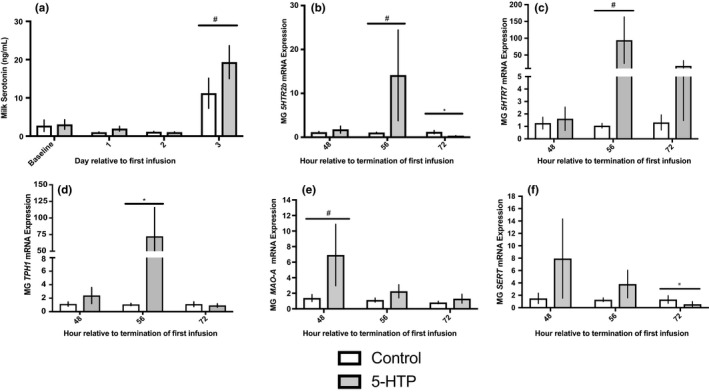
Milk serotonin concentrations (a) (*n* = 6) and mammary gland mRNA expression of *5HTR2b* (b) (5‐HTP *n* = 6, *n* = 6, *n* = 5; Control *n* = 5, *n* = 4, *n* = 4), *5HTR7* (c) (5‐HTP *n* = 6, *n* = 6, *n* = 5; Control *n* = 4, *n* = 4, *n* = 3), *TPH1* (d) (5‐HTP *n* = 6, *n* = 5, *n* = 5; Control *n* = 6, *n* = 4, *n* = 3), *MAO*‐*A* (e) (5‐HTP *n* = 6, *n* = 6, *n* = 6; Control *n* = 5, *n* = 5, *n* = 5) and *SERT* (f) (5‐HTP *n* = 6, *n* = 6, *n* = 5; Control *n* = 4, *n* = 6, *n* = 4) in multiparous Holstein dairy cows receiving 1 L of 1.5 mg/kg 5‐HTP dissolved in saline or 1 L of saline solution (Control = Saline) for three consecutive days. Statistics were performed using PROC MIXED procedure with repeated measures. Transformed data presented as means ± SEM. Milk serotonin concentrations: day (*p* < 0.0001). *5HTR2b* mRNA: time (*p* = 0.01) and treatment*time (*p* = 0.04). *SERT* mRNA: time (*p* = 0.08) and treatment*time (*p* = 0.08). Asterisks denote statistical significance between groups (*p < 0.05 and #0.10 < *p* > 0.05)

Across the experimental period milk serotonin concentrations were numerically increased in 5‐HTP infused cows (*p* = 0.22; Figure 5a). A day effect was observed for milk serotonin concentrations (*p* < 0.0001), with increased milk serotonin occurring on d3 in both the control and 5‐HTP treated cows; however, within d3 5‐HTP infused cows exhibited increased milk serotonin concentrations relative to control (*p* = 0.09). Serotonin receptor and metabolic genes were also impacted, with a tendency for increased mRNA expression of *5HTR2b* and *5HTR7* 8 h (56 h) after termination of the final infusion (*p* = 0.07 and *p* = 0.10, respectively; Figure 5b and c). Following a similar time pattern, 8 h (56 h) after termination of final infusion 5‐HTP treated cows had an approximate 70‐fold increase in *TPH1* mRNA expression compared to control cows (*p* = 0.04; Figure 5d). *MAO*‐*A* mRNA tended to be increased (*p* = 0.08; Figure 5e) immediately after termination of the final infusion (48 h). Cows infused with 5‐HTP had decreased expression of *SERT* mRNA 24 h (72 h) after termination of the final infusion (*p* = 0.03; Figure 5f).

### 3.6 Mammary serotonin and calcium tissue concentrations were altered in response to 5‐HTP infusion

**FIGURE 6:**
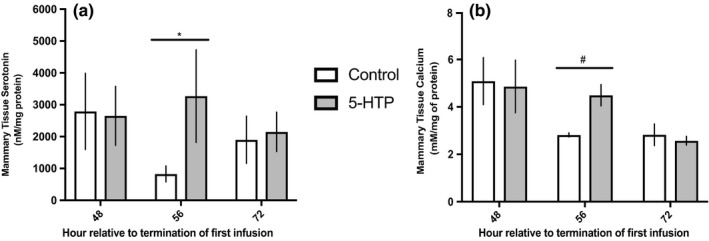
Mammary tissue serotonin concentrations (a) (*n* = 6) and mammary tissue calcium concentrations (b) (5‐HTP *n* = 5, *n* = 6, *n* = 4; Control *n* = 6, *n* = 5, *n* = 5) in multiparous Holstein dairy cows receiving 1 L of 1.5 mg/kg 5‐HTP dissolved in saline or 1 L of saline solution (Control = Saline) for three consecutive days. Statistics were performed using PROC MIXED procedure with repeated measures. Transformed data presented as mean ± SEM. Mammary tissue serotonin concentrations: treatment (*p* = 0.07). Mammary tissue calcium concentrations: time (*p* = 0.004). Asterisks denote statistical significance between groups (*p < 0.05 and ^#^0.10 < *p* > 0.05)

Due to observed systemic and milk changes in serotonin and calcium, we evaluated mammary serotonin and calcium content. Infusion of 5‐HTP increased (*p* = 0.07) mammary serotonin content in the mammary gland, with a significant 2000 nM/mg of protein increase in serotonin content 8 h (56 h) after termination of the third infusion compared to control cows (*p* = 0.04; Figure 6a). Next, we evaluated mammary calcium content, and while no differences were observed across the experimental period, a tendency for increased mammary calcium content also occurred 8 h (56 h) after termination of the third infusion in 5‐HTP treated cows (*p* = 0.09; Figure 6b).

## 4 DISCUSSION

The data within illustrates a novel coordinated response to 5‐HTP infusion at the local level of the mammary gland on calcium transport and serotonin metabolism and supports 5‐HTP’s ability to illicit a transient hypocalcemia, instigating the negative feedback mechanisms critical for calcium homeostasis at the onset of lactation. This further adds understanding to the biology underpinning serotonin's potential as an innovative new strategy for mitigating hypocalcemia in the dairy cow, a devasting disease for cow health and productivity (Goff, 2014). Collectively, the data herein demonstrate a coordination of endocrine and autocrine/paracrine mechanisms by which serotonin may govern calcium homeostasis in the dairy cow.

Infusion of 5‐HTP increased circulating serotonin concentrations, with a measurable increase occurring on d1 of the experiment, corroborating previous work showing 2 days of infusions of 5‐HTP are necessary to stimulate a robust increase in blood serotonin (Connelly et al., 2020; Laporta et al., 2015; Marrero et al., 2019). Additionally, highest circulating serotonin concentrations occurred 56 h after termination of first infusion, with elevation of circulating serotonin persisting 24 h post final 5‐HTP administration. Maintenance in elevation of blood serotonin may be attributed to the majority of peripheral serotonin, ~95% of whole body serotonin, being stored within platelets, which have a minimum half‐life of 3 days depending on species (Osim & Wyllie, 1983; Welford et al., 2016). Furthermore, supraphysiological serotonin action has been suggested to alter milk secretion (Horseman and Collier, 2014; Stull et al., 2007). Milk yield declined in 5‐HTP treated cows, but no treatment differences were observed in milk yield data and histological findings. These data reiterate previous work demonstrating 5‐HTP does not inhibit the dairy cow's ability to maintain lactational competency (Hernandez‐Castellano et al., 2017; Laporta et al., 2015; Slater et al., 2018; Weaver et al., 2016), but should be further explored due to the link between serotonin and milk stasis.

Blood calcium concentrations decreased in cows infused with 5‐HTP across the experiment, supporting a previous finding of a transient decline in blood calcium immediately after 5‐HTP infusion (Laporta et al., 2015). The decline in calcium concentrations in the current study fell below 2.0 mM, with steeper subsequent blood calcium decreases each ensuing day. Despite a lack of a collective definition of subclinical hypocalcemia, diagnostic thresholds used for subclinical calcium concentrations have ranged from 2.0–2.2 mM (Martinez et al., 2012; Rodriguez et al., 2017; Venjakob et al., 2018). Interestingly, the decline in blood calcium on d0 of 5‐HTP treatment occurred when circulating serotonin and PTH were not increased. While transient hypocalcemia seems counter intuitive in improving hypocalcemic incidences, calcium is homeostatically regulated by negative feedback mechanisms. Dairy cows that experience a transient hypocalcemia in the immediate period post‐partum have been shown to produce more milk, suggesting an improved adaptation to lactation facilitated by transient hypocalcemia (McArt & Neves, 2020). Decreases in calcium concentrations during lactation are believed to trigger PTHrP production in the mammary gland, allowing PTHrP to potentiate bone calcium mobilization to liberate calcium for maternal and mammary pools (Ryan & Kovacs, 2019). Although PTH is a crucial calciotropic hormone, mammary‐derived PTHrP is believed to play a more dominant role in calcium homeostasis during lactation (Kovacs, 2005).

Serotonin is thought to potentiate bone mobilization through increased mammary‐derived PTHrP (Hernandez et al., 2012). Cows infused with 5‐HTP exhibited an up‐regulation of *PTHLH* mRNA 56 h after termination of the first infusion, which coincided with up‐regulation of *CASR* mRNA. Serotonin has been shown to modulate *PTHLH* and *CASR* mRNA expression in the mammary gland during lactation to coordinate calcium metabolism and bone mobilization (Hernandez et al., 2012; Laporta, Keil, Vezina et al., 2014; Laporta et al., 2013). Interestingly, this increase in *PTHLH* expression occurred when blood serotonin concentrations were also highest in 5‐HTP infused cows. Additionally, CASR is considered the ultimate conductor for calcium metabolism, systemically and at the level of the mammary gland, to aid in coordination of calcium transport into milk (VanHouten et al., 2007). Coinciding increases in mRNA of *CASR* and *PTHLH* in the mammary gland of cows infused with 5‐HTP suggests a feedback loop that may be stimulated by serotonin and modulate systemic and mammary calcium dynamics. Additionally, increased *PTHLH* mRNA and lack of response in plasma PTH further suggests serotonin's regulation of calcium is outside of the classical PTH‐pathway and may be PTHrP‐dependent. This recapitulates a previous finding in periparturient cows, with 5‐HTP administration eliciting no change in PTH concentrations, while control animals displayed the classical PTH peak at parturition (Hernandez‐Castellano et al., 2017).

While 5‐HTP infused cows had lower blood calcium concentrations, mRNA and protein of ORAI1 in the mammary of 5‐HTP cows were upregulated in an oscillating pattern. ORAI1, a store‐operated calcium channel, is considered to be an imperative entry point for calcium into MEC, working in coordination with other organelles within the MEC to generate, reload and maintain cellular calcium stores (Cross et al., 2014; Davis et al., 2015). Research in cultured coronary arteries treated with serotonin demonstrated alterations in ORAI1, suggesting ORAI1 may be a participant in serotonin's calcium regulation (Deng et al., 2014). Further, a tendency in elevated mammary calcium tissue content corresponded with timing of mRNA and protein changes of ORAI1 in the 8 h after termination of the final infusion. Interestingly, a down‐regulation in *SERCA* mRNA, an intracellular calcium transport ATPase, occurred 16 h later. Mammary calcium flux is meticulously regulated due to cellular toxicity and the importance in maintenance of calcium homeostasis within the MEC. Many mechanisms are in place to support and respond to accumulations in tissue calcium, with store operated calcium channels often working in coordination with one another (Cross et al., 2014; Reinhardt & Horst, 1999). We did not hypothesize an increase in mammary gland calcium content due to the rigorous regulation of cellular calcium, and to our knowledge this is the first study showing changes in tissue calcium content in tandem with milk calcium and mammary calcium transport post 5‐HTP treatment. Mirrored time changes in *ORAI1* mRNA, tissue serotonin as well as tissue calcium could support the increase in milk calcium in 5‐HTP infused cows shown in the current study and previous research as serotonin modulates mammary calcium transport (Laporta et al., 2015). While the basolateral calcium MEC transporter ORAI1 was increased transcriptionally and translationally, the apical MEC transporter *PMCA2* and other intracellular calcium regulators (*SPCA1*, *NCX1* and *SERCA2)* were not increased transcriptionally. These transporters are essential for MEC calcium homeostasis and is contradictory to previous research with 5‐HTP administration to rodents (Laporta, Keil, Vezina et al., 2014; Laporta et al., 2013).

Circulating serotonin and mammary serotonin content were increased in cows infused with 5‐HTP, with the highest blood serotonin concentrations coinciding with an increase in mammary serotonin content 56 h after termination of first infusion. These changes were accompanied with increased *TPH1* mRNA, the enzyme that catalyzes the reaction to synthesize 5‐HTP, at the same timepoint. Surprisingly, no changes in *SERT* mRNA occurred. The lack of response in *SERT* expression may be attributed to SERT acting biphasically in response to different serotonin exposure levels (Mercado & Kilic, 2010). Expression of *MAO*‐*A*, the primary form of the enzyme responsible for metabolizing serotonin, was upregulated immediately after termination of the final infusion. These findings may indicate an overall change in mammary serotonin metabolism as the tissue is responding to changes in precursor supply, uptake and degradation while stabilizing tissue serotonin content (Bonnin & Levitt, 2011). Despite no increases in *SERT* mRNA, serotonin receptors *5HTR2b* and *5HTR7* had similar increases in transcription relative to control cows 8 h after termination of the final infusion (56 h relative to termination of first infusion), analogous with timing of tissue serotonin, tissue calcium, *ORAI1*, *PTHLH* and *CASR* mRNA expression changes. Of note, serotonin's modulation of calcium metabolism and *PTHLH* has been demonstrated to potentially act via the 5HTR2b receptor (Laporta, Keil, Vezina et al., 2014), but 5HTR7 is also expressed and conserved across humans, cattle and mice (Hernandez et al., 2009). In rodents, exogenous administration of 5‐HTP to TPH1^−/−^ dams fully rescued *5HTR2b* expression but did not fully recover the WT phenotype of 5HT2b downstream signaling pathways (Laporta, Keil, Vezina et al., 2014). Interestingly, ORAI1 changes occurred in tandem with alterations in 5HTR2a in cultured coronary arteries and shares similarity in signaling to 5HTR2b (Deng et al., 2014). Research has focused on various serotonin receptor actions in response to 5‐HTP administration, with their action non‐neuronally a major gap yet to be filled. Suggesting, in addition to data presented here, other serotonin receptors may need to be evaluated during mammary serotoninergic axis changes. Collectively, mammary serotonin metabolism appears to be stimulated in response to 5‐HTP infusion, with additional precursor presumably being supplied to allow for changes in mammary serotonin signaling and metabolism driving alterations in mammary calcium trafficking.

This experiment recapitulates that treatment with 5‐HTP induces an immediate transient hypocalcemia and reveals potential timing of endocrine and mammary autocrine/paracrine action in response to 5‐HTP administration. More interestingly, the data herein is the first to illustrate corresponding mammary serotonin and calcium changes in a uniquely coordinated fashion in the 8 h after termination of 5‐HTP infusion. This study further demonstrates the need for a clearer understanding of non‐neuronal receptor dynamics in the mammary gland to determine primary regulation of the serotonin‐calcium axis. Collectively, these data suggest the existence of a coordinated serotonin‐calcium feedback loop involving endocrine and autocrine/paracrine mechanisms that regulate maternal and mammary calcium homeostasis.

## CONFLICT OF INTEREST

The authors declare no conflict of interest.

## AUTHOR CONTRIBUTION

MKC, SRW and LLH designed this experiment. MK, HPF and JMK assisted in animal care and experiment conduction. MKC and LLH wrote and analyzed the experiment.
